# Supplementary material for: Trying to use temporal and kinematic parameters for the classification in wheelchair badminton
Source: PLoS One. 2025 Mar 28;20(3):e0315939. doi: 10.1371/journal.pone.0315939 (PMC11952265; doi:10.1371/journal.pone.0315939)
Supplement: Attachment — Submitted filename: PONE-D-24-26194_reviewer.pdf [file pone.0315939.s002.pdf]

PLOS ONE

Trying to use spatiotemporal parameters for the classification in wheelchair badminton.

--Manuscript Draft--

**Manuscript Number:**	PONE-D-24-26194
**Article Type:**	Research Article
**Full Title:**	Trying to use spatiotemporal parameters for the classification in wheelchair badminton.
**Short Title:**	Spatiotemporal parameters and classification in wheelchair badminton.
**Corresponding Author:**	Ilona Alberca, Student<br>Universite de Toulon<br>Toulon, FRANCE
**Keywords:**	Wheelchair; badminton; Biomechanics; Classification; performance; cluster
**Abstract:**	Introduction: This study explores the potential for the spatiotemporal data link to propulsion technique and athlete performance collected here to contribute to evidence-based classification for wheelchair badminton athletes. Materials and Methods: Nineteen experienced wheelchair badminton players underwent propulsion tests with a badminton racket. Wheelchair were equipped with inertial measurement units. The first analysis conducted involved comparing the parameters between WH1 and WH2. Subsequently, a hierarchical clustering analysis was performed on the parameters with significant differences. Results: Regarding propulsion technique parameters, WH1 athletes exhibit a longer braking phase compared to WH2 athletes. Generally, the performance of WH1 athletes is inferior to that of WH2 athletes. Concerning hierarchical clustering analysis, the results reveal the formation of three clusters based on principal components explaining 70% of the variation in the parameters considered in the analysis. Conclusion: Thus, the results of this study indicate a longer braking time for WH1 athletes compared to WH2, along with lower overall performance. The clusters results could suggest a potential evolution of the current classification towards three distinct classes of wheelchair badminton players. However, these findings should be interpreted with caution, given that the included performance parameters can be influenced by numerous factors, potentially undermining the robustness of the clustering methodology employed. This study highlights the need to strengthen the current classification process in wheelchair badminton.
**Order of Authors:**	Ilona Alberca, Student
	Bruno Watier
	Félix Chénier
	Brassart Florian
	Jean-Marc Vallier
	Arnaud Faupin
**Additional Information:**	
**Question**	**Response**
**Financial Disclosure**<br><br>Enter a financial disclosure statement that describes the sources of funding for the work included in this submission. Review the submission guidelines for detailed requirements. View published research articles from *PLOS ONE* for specific examples.	Yes

Powered by Editorial Manager® and ProduXion Manager® from Aries Systems Corporation

This statement is required for submission and **will appear in the published article** if the submission is accepted. Please make sure it is accurate.<br><br>**Funded studies**<br>Enter a statement with the following details:<br>• Initials of the authors who received each award<br>• Grant numbers awarded to each author<br>• The full name of each funder<br>• URL of each funder website<br>• Did the sponsors or funders play any role in the study design, data collection and analysis, decision to publish, or preparation of the manuscript?<br><br>Did you receive funding for this work?	
Please add funding details.<br>as follow-up to "**Financial Disclosure**<br><br>Enter a financial disclosure statement that describes the sources of funding for the work included in this submission. Review the submission guidelines for detailed requirements. View published research articles from *PLOS ONE* for specific examples.<br><br>This statement is required for submission and **will appear in the published article** if the submission is accepted. Please make sure it is accurate.<br><br>**Funded studies**<br>Enter a statement with the following details:<br>• Initials of the authors who received each award<br>• Grant numbers awarded to each author<br>• The full name of each funder<br>• URL of each funder website<br>• Did the sponsors or funders play any role in the study design, data collection and analysis, decision to publish, or preparation of the manuscript?<br><br>Did you receive funding for this work?"	This work was supported by a French government grant, managed by the Agence Nationale de la Recherche (ANR) under the "France 2030" program, reference ANR-19-STHP-0005.

Please select the country of your main research funder (please select carefully as in some cases this is used in fee calculation).<br>as follow-up to "**Financial Disclosure**<br><br>Enter a financial disclosure statement that describes the sources of funding for the work included in this submission. Review the submission guidelines for detailed requirements. View published research articles from *PLOS ONE* for specific examples.<br><br>This statement is required for submission and **will appear in the published article** if the submission is accepted. Please make sure it is accurate.<br><br>**Funded studies**<br>Enter a statement with the following details:<br>• Initials of the authors who received each award<br>• Grant numbers awarded to each author<br>• The full name of each funder<br>• URL of each funder website<br>• Did the sponsors or funders play any role in the study design, data collection and analysis, decision to publish, or preparation of the manuscript?<br><br>Did you receive funding for this work?"	FRANCE - FR
**Competing Interests**<br><br>Use the instructions below to enter a competing interest statement for this submission. On behalf of all authors, disclose any competing interests that could be perceived to bias this work—acknowledging all financial support and any other relevant financial or non-financial competing interests.<br><br>This statement is required for submission and **will appear in the published article** if the submission is accepted. Please make sure it is accurate and that any funding	NO authors have competing interests

sources listed in your Funding Information later in the submission form are also declared in your Financial Disclosure statement.<br><br>View published research articles from *PLOS ONE* for specific examples.<br><br>**NO authors have competing interests**<br><br>Enter: *The authors have declared that no competing interests exist.*<br><br>**Authors with competing interests**<br><br>Enter competing interest details beginning with this statement:<br><br>*I have read the journal's policy and the authors of this manuscript have the following competing interests: [insert competing interests here]*<br><br>* typeset	
**Ethics Statement**<br><br>Enter an ethics statement for this submission. This statement is required if the study involved:<br><br>• Human participants<br>• Human specimens or tissue<br>• Vertebrate animals or cephalopods<br>• Vertebrate embryos or tissues<br>• Field research<br><br>Write "N/A" if the submission does not require an ethics statement.<br><br>General guidance is provided below. Consult the submission guidelines for detailed instructions. **Make sure that all information entered here is included in the Methods section of the manuscript.**	The data of this study were collected during the French championships of Nueil-les-Aubiers from 14 to 16 January 2022 and Saint-Orens from 13 to 15 January 2023. The experimental protocol was approved by the Comité d'Ethique pour les Recherches en STAPS (CERSTAPS) from Conseil National des Universités de France [certificate #CERSTAPS IRB00012476-2021-11-06-274] filed on February 2021 and accepted on Jun 2021. Participants were recruited starting on December 1st, 2021, and end on January 10, 2022. All participants have received and signed a written informed consent and information notice.

**Format for specific study types**<br><br>**Human Subject Research (involving human participants and/or tissue)**<br>• Give the name of the institutional review board or ethics committee that approved the study<br>• Include the approval number and/or a statement indicating approval of this research<br>• Indicate the form of consent obtained (written/oral) or the reason that consent was not obtained (e.g. the data were analyzed anonymously)<br><br>**Animal Research (involving vertebrate animals, embryos or tissues)**<br>• Provide the name of the Institutional Animal Care and Use Committee (IACUC) or other relevant ethics board that reviewed the study protocol, and indicate whether they approved this research or granted a formal waiver of ethical approval<br>• Include an approval number if one was obtained<br>• If the study involved *non-human primates*, add *additional details* about animal welfare and steps taken to ameliorate suffering<br>• If anesthesia, euthanasia, or any kind of animal sacrifice is part of the study, include briefly which substances and/or methods were applied<br><br>**Field Research**<br><br>Include the following details if this study involves the collection of plant, animal, or other materials from a natural setting:<br>• Field permit number<br>• Name of the institution or relevant body that granted permission	
**Data Availability**<br><br>Authors are required to make all data underlying the findings described fully available, without restriction, and from the time of publication. PLOS allows rare exceptions to address legal and ethical concerns. See the PLOS Data Policy and FAQ for detailed information.	Yes - all data are fully available without restriction

A Data Availability Statement describing where the data can be found is required at submission. Your answers to this question constitute the Data Availability Statement and **will be published in the article**, if accepted.<br><br>**Important:** Stating 'data available on request from the author' is not sufficient. If your data are only available upon request, select 'No' for the first question and explain your exceptional situation in the text box.<br><br>Do the authors confirm that all data underlying the findings described in their manuscript are fully available without restriction?	
**Describe where the data may be found in full sentences. If you are copying our sample text, replace any instances of XXX with the appropriate details.**<br><br>• If the data are **held or will be held in a public repository**, include URLs, accession numbers or DOIs. If this information will only be available after acceptance, indicate this by ticking the box below. For example: *All XXX files are available from the XXX database (accession number(s) XXX, XXX.).*<br>• If the data are all contained **within the manuscript and/or Supporting Information files**, enter the following: *All relevant data are within the manuscript and its Supporting Information files.*<br>• If neither of these applies but you are able to provide **details of access elsewhere**, with or without limitations, please do so. For example:<br><br>*Data cannot be shared publicly because of [XXX]. Data are available from the XXX Institutional Data Access / Ethics Committee (contact via XXX) for researchers who meet the criteria for access to confidential data.*<br><br>*The data underlying the results presented in the study are available from (include the name of the third party*	All relevant data are within the manuscript and its Supporting Information files.

and contact information or URL).<br>• This text is appropriate if the data are owned by a third party and authors do not have permission to share the data.<br><br>* typeset	
Additional data availability information:	

Powered by Editorial Manager® and ProduXion Manager® from Aries Systems Corporation

Manuscript Click here to access/download;Manuscript;Manuscript.docx

Trying to use spatiotemporal parameters for the classification in wheelchair badminton.

Spatiotemporal parameters and classification in wheelchair badminton.

Ilona Alberca[1*], Bruno Watier [2,3], Félix Chénier[4,5], Florian Brassart[1], Jean-Marc Vallier[1], and Arnaud Faupin[1]

[1]Laboratoire IAPS, Université de Toulon, La Garde, France ; [2]LAAS-CNRS, Université de Toulouse, CNRS, UPS, Toulouse, France ; [3]CNRS-AIST JRL (Joint Robotics Laboratory), IRL, National Institute of Advanced Industrial Science and Technology (AIST), 1-1-1 Umezono, 305-8560 Tsukuba, Ibaraki, Japan ; [4]Mobility and Adaptive Sports Research Lab, Department of Physical Activity Sciences, Université du Québec à Montréal, Montreal, QC, Canada, [5]Centre for Interdisciplinary Research in Rehabilitation of Greater Montreal, Institut Universitaire sur la Réadaptation en Déficience Physique de Montréal, Montreal, QC, Canada

*: corresponding author; ilona.alberca@univ-tln.fr

Abstract:

Introduction: This study explores the potential for the spatiotemporal data link to propulsion technique and athlete performance collected here to contribute to evidence-based classification for wheelchair badminton athletes. **Materials and Methods**: Nineteen experienced wheelchair badminton players underwent propulsion tests with a badminton racket. Wheelchair were equipped with inertial measurement units. The first analysis conducted involved comparing the parameters between WH1 and WH2. Subsequently, a hierarchical clustering analysis was performed on the parameters with significant differences. **Results:** Regarding propulsion technique parameters, WH1 athletes exhibit a longer braking phase compared to WH2 athletes. Generally, the performance of WH1 athletes is inferior to that of WH2 athletes. Concerning hierarchical clustering analysis, the results reveal the formation of three clusters based on principal components explaining 70% of the variation in the parameters considered in the analysis. **Conclusion:** Thus, the results of this study indicate a longer braking time for WH1 athletes compared to WH2, along with lower overall performance. The clusters results could suggest a potential evolution of the current classification towards three distinct classes of wheelchair badminton players. However, these findings should be interpreted with caution, given that the included performance parameters can be influenced by numerous factors, potentially undermining the robustness of the clustering methodology employed. This study highlights the need to strengthen the current classification process in wheelchair badminton.

Key words: wheelchair; badminton; biomechanics; classification; impairment; cluster

Introduction

Classification of athletes is paramount in wheelchair sports, including badminton, aiming to allocate athletes into appropriate sport classes to minimize the influence of impairment on competition outcomes while prioritizing sporting excellence (1). As emphasized by Goosey-Tolfrey and Leitch (2), classification reflects the individual's level of disability. Following the functional classification system proposed by Strohkendl in 1982, endorsed by the International Wheelchair Basketball Federation, wheelchair badminton employs a similar approach, assessing athletes' functional abilities for classification purposes (3). Specifically, they are classified into two categories: WH1 and WH2. WH1 participants are manual wheelchair users with abdominal and lower limb paralysis, while WH2 participants possess abdominal capabilities but experience lower limb paralysis with partial sensation (4).

For individuals with spinal cord injuries, impairments are directly link to the level of the lesion, significantly affecting postural stability and propulsion abilities, especially in the abdominal region (5,6). Indeed, postural stability, commonly referred to as sitting balance, is compromised during the execution of a task such as wheelchair propulsion due to the loss of activity in certain muscles, such as the erector spinae, for example (7). Therefore, differences in performance between the WH1 and WH2 classes can be expected due to disparities in handicap levels and indeed, three studies have revealed differences in match intensity and shots played between WH1 and WH2 (8–10). However, none have investigated the impact of classification on spatiotemporal parameters linked to propulsion technique and athlete performance.

Given the novelty of the sport, wheelchair badminton classification was recently established by the International Paralympic Committee Athlete Classification Code in 2015. The classification process is initiated by determining eligibility based on a minimal level of impairment. Subsequently, a physical and technical evaluation is conducted to differentiate

between the WH1 and WH2 classifications (3). This physical and technical evaluation take place during a tournament or training match where assessors are asked to visually identify the following profiles:

- WH1: "Players demonstrate functional limitation based on muscle power and range of motion of the trunk and possibly upper limbs during a match or training session." (3).
- WH2: "Players have a functional limitation based on limited muscle power or range of motion, requiring the use of walking aids. A shift in the center of gravity may result in loss of balance, for example when attempting to pivot or stop and start." (3).

However, this classification process does not involve any objective measurement of the athletes' performance capabilities. The International Paralympic Committee (IPC) has established the conceptual framework of evidence-based classification for several years (11). Evidence-based classification indicates that each sport should develop its own classification system based on empirical evidence demonstrating the association between impairment and performance in that sport. Following this rationale, the use of spatiotemporal parameters related to wheelchair badminton propulsion technique and player performance could allow for the inclusion of this discipline within the framework of evidence-based classification. Notably, the integration of inertial measurement units (IMUs) has recently facilitated the acquisition of on-field performance data for wheelchair athletes (12). In a manuscript in pre-print and currently under submission, Alberca et al. (13) recently utilized two IMUs to assess diverse performance parameters among wheelchair badminton players during a one-minute field test.

Hence, the main objective of this study is to explore the potential for the spatiotemporal data link to propulsion technique and athlete performance collected here to contribute to evidence-based classification for wheelchair badminton athletes. To meet this main objective, it will first be necessary to examine whether classification has an impact on these same

spatiotemporal parameters. Previous studies lack substantial evidence to formulate hypotheses regarding spatiotemporal differences between the WH1 and WH2 divisions. However, given their classification based on impairment level, it can be hypothesized that WH1 athletes with more severe functional limitations will demonstrate spatiotemporal patterns indicative of lower performance compared to WH2 athletes. Additionally, it is hypothesized that the spatiotemporal parameters measured in this study correctly discriminate the two classifications of wheelchair badminton athlete.

Materials and methods

Study design

This study aims to explore the possibility that the spatiotemporal data representative of propulsion technique and athlete performance from this study could serve as evidence for the evidence-based classification. The same protocol as in the study by Alberca et al. (13) was used in this study. Warmed-up participants performed consecutive forward and backward sprints over 3 meters for 1 minute to carry out a test as close as possible to match conditions as shown in Figure 1. The duration of 1 min and the length of 3 m of the test were chosen to correspond to the characteristics of this sport without inducing too intense an effort for the athletes (court size: 3.96m long and effective playing time: 5.7 min and 7.7 min) (9).

[Figure 1]

Figure 1: Schematic diagram of forward/backward propulsion test

During the experiment, all athletes started from a stationary position at the 3-meter line, demarcated by cones, initiating in forward propulsion. Upon the starting signal, they sprinted forward along the 3-meter line until reaching the end. Subsequently, they braked and proceeded in backward propulsion along the same course, repeating this sequence for 1 minute. When switching between propulsion directions, athletes were required to pass the large wheels of their

wheelchairs beyond the cones at each end of the track. One trial was conducted for each participant with their own racket and personal wheelchair. Each athlete held the racket on their preferred side, referred to as the racket side. A 5-minute break was observed between each trial. Although the propulsion technique was not prescribed, upon observation, all athletes employed synchronous propulsion.

Ethics

The data of this study were collected during the French championships of Nueil-les-Aubiers from 14 to 16 January 2022 and Saint-Orens from 13 to 15 January 2023. The experimental protocol was approved by the Comité d'Ethique pour les Recherches en STAPS (CERSTAPS) from Conseil National des Universités de France [certificate #CERSTAPS **IRB00012476-2021-11-06-274**] filed on February 2021 and accepted on Jun 2021. Participants were recruited starting on December 1st, 2021, and end on January 10, 2022. All participants have received and signed a written informed consent and information notice.

Participants

A total of 19 wheelchair badminton athletes was included in this study. Inclusion criteria required participants to be at a national level or higher in wheelchair badminton and have a minimum of one year of experience in playing the sport. Participants were excluded if they reported any pain or injury that could hinder their ability to propel their wheelchair. To determine the minimum sample size required for this study, a statistical power test has been made with de Rhodes et al. (14) as the reference article. The required sample size was estimated at N = 16 participants. Considering this result, a total of N = 19 badminton athletes was included in this analysis. Statistical power testing was performed using G*Power software (G*Power, 2020; g-power.apponic.com). Characteristics of all participants are presented in Table 1.

	Gender	Class	Age (years)	Height (cm)	Body mass (kg)	BMI (kg/m2)	Years of practice (years)	Racket side	Pathology	Camber (°)	Wheel size (inch)
1	Female	WH2	55	162	60	37.0	9	R	Paraplegia	20	24
2	Female	WH1	45	165	58	35.2	10	R	Paraplegia	18	25
3	Male	WH2	31	180	60	33.3	6	L	Paraplegia	18	26
4	Male	WH1	37	176	67	38.1	6	R	Paraplegia	20	25
5	Male	WH1	45	158	64	40.5	17	R	Spinabifida	18	25
6	Male	WH2	48	187	75	40.1	9	R	Paraplegia	20	26
7	Female	WH1	53	171	68	39.8	8	R	Paraplegia	20	25
8	Male	WH1	45	168	71	42.3	12	R	Paraplegia	20	25
9	Female	WH1	33	165	60	36.4	2	R	Paraplegia	20	24
10	Female	WH2	22	135	43	31.9	6	R	Osteogenesis imperfecta	18	24
11	Male	WH2	38	185	63	34.1	2	R	Paraplegia	20	25
12	Male	WH2	44	165	58	35.2	9	R	Paraplegia	18	25
13	Male	WH1	40	187	70	37.4	5	R	Paraplegia	20	25
14	Male	WH1	49	185	94	50.8	5	R	Paraplegia	20	25
15	Male	WH1	52	160	60	37.5	3	L	Polyomielitis	20	25
16	Female	WH2	41	175	68	38.9	9	R	Incomplete paraplegia	18	25
17	Female	WH2	37	170	60	35.3	3	R	Incomplete paraplegia	20	25
18	Female	WH2	27	156	47	30.1	4	R	Algoneuro dystrophy	20	25
19	Male	WH1	33	178	100	56.2	14	R	Paraplegia	20	25
Mean (SD)			40.8(8.8)	169.9(12.7)	65.6(13.1)	38.4(6.0)	7.3(4.0)				

Table 1: Participants' characteristics.

With SD: standard deviation; BMI: Body Mass Index.

Data measurement

Inertial measurement units (IMUs) were used to collect on-field data (12,15–17). Their wheelchair was equipped bilaterally with two IMUs (128 Hz, 3*3: accelerometer, gyroscope, magnetometer, and Bluetooth module, WheelPerf System, AtoutNovation, France). IMUs were placed on each wheel hub, and the gyroscope was used to estimate the direct rotational velocity of the wheel around the z-axis, considering the camber angle of the wheelchair as indicated by Fuss et al. (18). The z-axes of gyroscopes were placed perpendicularly to the wheel planes (19).

The data were filtered using a Butterworth low-pass filter of order 2 with a cutoff frequency of 4 Hz.

Data were processed using Python 3.11 and Kinetics Toolkit 0.11 (20). Figure 1 shows an example of propulsion velocity curve for one sprint in forward and backward propulsion. The various phases visible in Figure 1, such as acceleration, deceleration, or the transition phases, were manually marked using events and enabled us to calculate the outcome parameters. All the outcome parameters were calculated for all the 3 meters sprints performed by the athletes.

Outcome Parameters

To meet the objectives of this article, the same propulsion technique and performance parameters as in the study by Alberca et al. (13) were used and are listed in Table 2.

**Parameters**	**Description**
**Propulsion technique parameters**	
Propulsion phase time **(PP_{mean})** [s]	Time between the sprint start and the first peak velocity
Deceleration phase time **(DP_{mean})** [s]	Time between the last peak velocity and the sprint end
**Performance parameters**	
Sprint time **(ST_{mean})** [s]	Sprint time of each direction of propulsion
Transition time **(TT_{mean})** [s]	Time between the end of the deceleration phase and the start of the next sprint
Maximum velocity **(V_{max})** [m/s]	Maximum velocities reached on all sprint
Mean velocity **(V_{mean})** [m/s]	Mean velocities reached on all sprint
Peak velocity **(V_{peak})** [m/s]	First maximum velocity reached during the sprint
Acceleration **(A_{mean})** [m/s^2]	Mean acceleration between the sprint start and the first peak velocity
Deceleration **(D_{mean})** [m/s^2]	Mean deceleration between the last peak velocity and the sprint end

Table 2: Description of the outcome measures.

In addition to the definitions shown in Table 2, Figure 2 illustrates the various parameters calculated.

[Figure 2]

Figure 2: Example of propulsion velocity curve for one sprint in forward and backward propulsion

The acceleration and propulsion phase time were calculated only on the beginning of each sprint because it is the only moment when athletes accelerate the most from a stationary position since the wheelchair is stopped and has no velocity. The same reasoning is applied to the deceleration phase time and deceleration. These parameters are only calculated on the end of the sprint since it is the only moment when athletes brake to stop the wheelchair and completely decelerate (13).

Statistical methods

To meet the main objective of our study, the first step is to compare the data of WH1 and WH2 athletes in forward propulsion and in backward propulsion. To do this, the averaged IMU data for the left and right wheel were used, in forward and backward propulsion, for WH1 and WH2 athletes. Normality of the data was tested using a Shapiro-Wilk test, which showed that the data were not normally distributed. Therefore, non-parametric of Wilcoxon tests has been chosen for the comparisons. Significance was set at $p < 0.05$.

For each significant difference, the effect size *d* was calculated using the following equation:

$$d = \frac{mean(X_0) - mean(X_1)}{s.d.(X_0)}$$

With X: studied parameter, 0: data without racket according to the statistical analysis and 1: data with racket according to the statistical analysis.

Effect size was interpreted according to (21): small (d = 0.2), moderate (d = 0.5), and large (d = 0.8).

After this initial analysis, the main objective is to explore the possibility that the spatiotemporal data from this study could serve as evidence for the evidence-based classification of wheelchair badminton athletes. To this end, a principal component analysis (PCA) was performed on parameters showing a significant difference between WH1 and WH2 according to the previous statistical analysis. Propulsion technique parameters and performance parameters with no significant differences between WH1 and WH2 were not considered in the analysis. Then, hierarchical clustering was performed on the coordinates obtained through PCA to identify similarities across classifications. This clustering method is based on iteratively merging data into larger clusters based on their Euclidean distance. In this hierarchical clustering analysis, it is interesting to examine whether the clusters exhibit significant differences among them and to identify on which principal component these differences manifest between the clusters to enhance the understanding of the clusters. To test the hypothesis of a difference between clusters on each PCA component, two non-parametric Kruskal-Wallis tests were performed, as the normality hypothesis was rejected by the Shapiro-Wilk test. Then, pairwise comparisons of clusters on each PCA component were conducted using the Mann-Whitney post-hoc test with Bonferroni correction. Lastly, to understand the distribution of PC classifications within the clusters, a contingency table was created. Results are reported in percentage (%).

Results

Impact of classification on performance

The results of the comparison of data between WH1 and WH2 athletes are presented in Table 3 (a) for forward propulsion and Table 3 (b) for backward propulsion and, in Figure 3.

Table 3: Comparison of propulsion technique and performance parameters between the two classes (WH1 and WH2) for the forward propulsion (a) and the backward propulsion (b)

Forward	WH1	WH2	Comparison	
	**Mean(SD)**	**Mean(SD)**	***p***	**d**
	**Propulsion technique parameters**			
**PP_{mean} (s)**	0.47(±0.17)	0.44(±0.18)	0.376	0.076
**DP_{mean} (s)**	**0.45(±0.17)**	**0.37(±0.11)**	**0.002***	**0.263**
	**Performance parameters**			
**ST_{mean} (s)**	**2.07(±0.73)**	**2.19(±0.78)**	**<0.001**	**0.414**
**TT_{mean}[1] (s)**	0.39(±0.23)	0.41(±0.28)	0.806	0.021
**V_{max} (m/s)**	**4.31(±0.70)**	**4.71(±0.59)**	**<0.001***	**-0.332**
**V_{mean} (m/s)**	2.94(±0.53)	3.05(±0.42)	0.079	-0.150
**V_{peak} (m/s)**	**2.98(±0.94)**	**3.15(±0.89)**	**0.015***	**-0.208**
**A_{mean} (m/s^2)**	**5.36(±1.76)**	**6.08(±1.65)**	**<0.001***	**-0.300**
**D_{mean} (m/s^2)**	**8.46(±3.56)**	**11.77(±3.80)**	**<0.001***	**-0.482**

Backward	WH1	WH2	Comparison	
	**Mean(SD)**	**Mean(SD)**	***p***	**d**
	**Propulsion technique parameters**			
**PP_{mean} (s)**	0.43(±0.15)	0.43(±0.15)	0.483	0.060
**DP_{mean} (s)**	**0.43(±0.15)**	**0.35(±0.11)**	**<0.001***	**0.323**
	**Performance parameters**			
**ST_{mean} (s)**	**2.29(±0.63)**	**2.19(±0.78)**	**<0.001**	**0.453**
**TT_{mean}[2] (s)**	**0.43(±0.25)**	**0.41(±0.28)**	**<0.001**	**0.344**
**V_{max} (m/s)**	**3.84(±0.67)**	**4.17(±0.54)**	**<0.001***	**-0.347**
**V_{mean} (m/s)**	**2.64(±0.36)**	**2.83(±0.31)**	**<0.001***	**-0.321**
**V_{peak} (m/s)**	**2.66(±0.51)**	**3.05(±0.81)**	**<0.001***	**-0.292**
**A_{mean} (m/s^2)**	5.98(±1.63)	6.26(±1.55)	0.126	-0.131
**D_{mean} (m/s^2)**	**7.61(±2.63)**	**11.09(±3.46)**	**<0.001***	**-0.589**

With SD: standard deviation; p: p-value fixed at 0.05; d: effect size for the significant difference

Bold values indicate significant values.

[1]*: transition time from forward propulsion to backward propulsion;* [2]*: transition time from backward propulsion to forward propulsion.*

Regarding forward propulsion, WH1 athletes demonstrate slightly higher values for ST_{mean} and DP_{mean} compared to athletes in the WH2 class. Conversely, WH1 athletes exhibit slightly lower values for TT_{mean}, V_{max}, V_{peak}, A_{mean}, and D_{mean} in comparison to WH2 athletes. No significant difference was found regarding PP_{mean} and V_{mean} between WH1 and WH2 athletes.

In the initial analysis, significant disparities in velocity were observed between WH1 and WH2 athletes that may have large influence on TT_{mean}. Given TT_{mean}'s representation of 13 to 21% of the total sprint duration and is influenced by athletes' velocities, it was decided to exclude this parameter from the performance metrics considered for hierarchical clustering.

About backward propulsion, like forward propulsion, WH1 athletes exhibit significantly slightly higher values for ST_{mean} and DP_{mean} compared to WH2 athletes. Conversely, WH1 athletes show significantly lower values for TT_{mean}, V_{max}, V_{mean}, V_{peak}, and D_{mean} compared to WH2 athletes. No significant difference was found regarding PP_{mean} and A_{mean} between WH1 and WH2 athletes.

PCA and hierarchical clustering

The PCA revealed two principal components explaining together 64.35% of the overall variance. The first component (PC1) accounts for 54.30% of the variance and is linked to velocity parameters as indicated in Table 4. The second component (PC2) explains 10.05% of the variance and is related to temporal and propulsion technique parameters.

Table 4: Results of the principal component analysis and coordinate of each variable in each PCA components as well as percentage of variance

	Principal component 1 (PC1)	Principal component 2 (PC2)
Variance (%)	54.30	12.05
Forward propulsion		
DP_{mean}	-0.557	0.703
ST_{mean}	-0.791	0.468
V_{max}	0.850	0.293
V_{peak}	0.599	0.003
A_{mean}	0.727	-0.047
D_{mean}	0.772	-0.466
Backward propulsion		
DP_{mean}	-0.376	-0.242
ST_{mean}	-0.854	0.043
V_{max}	0.870	0.309
V_{mean}	0.822	0.436
V_{peak}	0.693	0.002
D_{mean}	0.768	0.288

Three distinct clusters were identified through hierarchical clustering analysis on PCA components. The three clusters are represented in Figure 3.

[Figure 3]

Figure 3: Clustering analysis in each variable on each PCA component.

With colored polygons: each cluster, colored dot: observations of each athlete in dark red for WH1 and blue for WH2

Regarding PC1, significant differences were noted across all clusters ($p < 0.001$). Regarding PC2, significant differences were noted for the cluster 1 ($p < 0.001$) and cluster 3 ($p = 0.002$). But no significant difference was observed for cluster 2 meaning that PC2 contribute contributes slightly less to the distinction between clusters than the PC1. These significant

differences make it possible to validate the discrimination of the clusters on the principal components identified. The percentage distribution of classifications by cluster is available in Table 5.

Table 5: Contingency table representing the percentages of WH1 and WH2 in each cluster (in %).

	WH1	WH2
Cluster 1	82.50	17.50
Cluster 2	45.26	54.74
Cluster 3	52.56	47.44

Cluster 1 is predominantly composed of WH1 athletes at 82.50% compared to 17.50% WH2. In contrast, the distributions of clusters 2 and 3 are much more balanced, with 45.26% WH1 and 54.74% WH2 for cluster 2, and 52.56% WH1 and 47.44% WH2 in cluster 3.

Discussion

To our knowledge, the analysis presented in this article represents the first exploration within the realm of wheelchair badminton. Its main objective of this study was to explore the utility of the spatiotemporal data link to propulsion technique and athlete performance to contribute evidence-based classification in wheelchair badminton. Differences in the parameters measured were observed between the two classifications in a way that indicates a longer braking time for WH1 compared to WH2, as well as overall lower performance. Contrary to our initial hypothesis, hierarchical clustering analysis did not align with current classifications, revealing three distinct clusters instead of two. This method is an attempt to allow the current classification system to evolve towards an evidence-based classification, by including objective scientific measurements of the performance of the athletes considered and their propulsion technique. Future research is needed to implement this method and its database.

Comparison of data between WH1 and WH2 athletes.

Regarding propulsion technique parameters, WH2 athletes exhibit a lower deceleration phase compared to those WH1, regardless of the direction of propulsion considered. This is in line with the initial hypothesis. However, the propulsion phase shows no significant differences between the two classes for both propulsion directions. It appears that athletes classified WH2 modify their propulsion technique by reducing braking phases. This observation could be attributed to the superior abdominal capabilities of WH2 athletes, allowing them to lean further forward or backward on the wheelchair compared to WH1 athletes, thereby enabling more effective braking.

Concerning performance parameters, transition time from one propulsion direction to another is shorter for WH1 compared to WH2, suggesting potentially superior temporal performance among WH1 athletes. This is at odds with the initial hypothesis. However, as previously stated, this outcome can be elucidated by the disparities in velocity between the two groups of athletes. Specifically, WH2 athletes, achieving a swifter conclusion to the sprint compared to WH1 athletes owing to their superior velocities, may consequently require more time for transitioning between propulsion directions. It is possible to assume that at imposed velocities, the results concerning the transition time could be different.

In forward propulsion, WH1 athletes exhibit a higher sprint time compared to WH2 athletes, as well as lower maximum and peak velocities, and lower acceleration and deceleration compared to WH2 athletes. Similar results are observed in backward propulsion, except for acceleration, which shows no significant differences between WH1 and WH2. This is in line with the initial hypothesis. These findings can be explained by their functional capacity, specifically abdominal strength, influencing trunk mobility. This variation in trunk mobility may account for the observed differences between WH1 and WH2. As per Vanlandewijck et al. (22), trunk movements are fundamental force-generating mechanisms in high-resistance

propulsion. These movements impact the rolling resistance of the athlete's wheelchair. However, increased rolling resistance can lead to reduced propulsion velocity, longer cycle and push times, increased mean power output, and propulsion forces (23–26). Trunk mobility also influences the orientation of the push angle on the handrim, a parameter linked to propulsion efficiency (22). It is plausible to assume that WH1 athletes, with limited abdominal capabilities, experience reduced trunk mobility. This limitation could negatively impact their rolling resistance and push angle, thereby reducing propulsion efficiency and, consequently, velocity, initial acceleration, and last deceleration. So, WH1 athletes may demonstrate lower significant performances compared to WH2.

Hierarchical clustering analysis

Cluster analysis on the principal components of the PCA revealed three clusters, contrary to the initial hypothesis of two expected clusters. According to the Mann-Whitney post-hoc test, only PC1 contributes significantly to distinguishing between clusters. PC1 primarily represents velocity data of athletes, which appears to be the most discriminative factor in categorizing athletes into three clusters. This finding suggests that the data from this study do not adequately classify athletes according to the two existing categories of wheelchair badminton. However, while the cluster analysis did not reveal two clusters, it revealed three. The distribution of athletes across the different clusters varies. In particular, the first cluster is predominantly composed of WH1 athletes (65.75%), while clusters 2 and 3 have much more balanced distributions between WH1 and WH2. To understand this distribution, the number of years of practice was studied as an explanatory factor. Athletes are categorized as “less experienced” if they have less than 5 years of practice, as “experienced” if they have between 5 and less than 10 years of experience, and as “very experienced” if they have 10 years or more of practice. Following this grouping, the proportions were determined in each cluster (see Table 6).

Table 6: Contingency table representing the percentages of beginners, intermediates and advanced in each cluster (in %).

	Less experienced	Experienced	Very experienced
Cluster 1	57.53	42.47	0.00
Cluster 2	46.97	50.00	3.03
Cluster 3	4.05	59.46	36.49

Based on the findings from Table 6, it is evident that cluster 1 predominantly consists of athletes labeled as "less experienced" (57.53%), while cluster 3 almost exclusively comprises "highly experienced" athletes. Additionally, cluster 2 presents a blend of athletes categorized as "less experienced" (46.97%) and "experienced" (50.00%). Consequently, years of experience emerge as a possible explanatory factor for the observed cluster patterns and the distribution of WH1 and WH2 athletes. These findings suggest that years of experience may exert an influence on athletes' performance and their propulsion technique.

The three clusters in this study would indicate a need to evolve the current classification towards a classification with 3 classes considering factors that are absent from the current process. However, the results of this study need to be interpreted with caution. Indeed, in this article, performance parameters were selected with propulsion technique parameters for clustering analysis due to their rapid measurement and ease of acquisition. However, it is important to acknowledge that an athlete's performance can vary for various reasons, both intrinsic and extrinsic. Taking these considerations into account, along with the results obtained in this study, which did not allow for the identification of the current classification, the decision to use performance parameters in the clustering analysis raises questions. Thus, the results of this article point more to the need to improve the current methodology employed, than to a revision of the current classification. One conceivable approach would be to incorporate functional parameters, such as trunk or upper limb mobility. In wheelchair basketball, for

example, functional classification assesses the trunk and upper limb capacities of players, reflecting their ability to perform various actions on the court. The validity of this approach has been demonstrated in various fields, including physiology, biomechanics, and game performance (2,27,28). Integrating similar objective biomechanical measures could strengthen the clustering methodology of this study and lead to a more precise classification based on tangible data. This evolution could enhance the classification process of wheelchair badminton players, or even prompt its revision if the results justify it. Moreover, such an approach would refine and scientifically reinforce the classification, bringing it closer to the concept of evidence-based classification.

Limitations

The main limitation of this article lies in the size of the population used for testing. Indeed, a cohort consisting of a limited number of WH1 and WH2 participants could potentially weaken the statistical models used for the performed cluster analysis. One potential avenue for improvement would be to enhance the existing database by including global players, thereby strengthening the existing data. With more data, it would be possible to strengthen the clusters obtained in the analysis.

Furthermore, the instruction given to athletes during the tests was to align the large wheelchair wheel with the start/finish lines to validate the sprint. Despite the particular attention paid to adhering to this instruction, it is possible that athletes did not consistently comply with it throughout the entire 1-minute test duration. Considering this, it is conceivable that the distance covered by each athlete may vary, potentially influencing the speed results. An improvement could involve employing photoelectric cells at the start and finish lines, signaled by an audible tone upon passage. Athletes would then be instructed to trigger the cell at the end of each sprint.

Perspectives

Regarding the characterization of WH1 and WH2 athletes, the results obtained in this article could serve as a performance database for coaches and athletes. Indeed, this could enable them to assess their position relative to a global average level, with the potential for individualization and strategic direction in athlete training.

A concrete perspective for the clustering results presented in this article would be to propose to the BWF to integrate more tests based on the evaluation of athletes' functional capacities and objective biomechanical measurements into the existing classification process. Indeed, with the aim of objectifying the classification process using rapid tests and non-invasive measurements, the current classification could become more representative of athletes' abilities, based on the concept of evidence-based classification. In addition to velocity measurements, it would be interesting to add the evaluation of the volume of action of athletes with measurements of trunk kinematics to the classification process.

Conclusion

To conclude, this article highlights the following points:

- WH1 athletes have a longer braking phase than WH2 athletes.
- Overall, WH1 athletes perform less well than WH2 athletes.
- The spatiotemporal data in this study did not allow for the identification of the current classification, as three clusters emerged instead of two.

Thus, the results of this study could suggest a potential evolution of the current classification towards three distinct classes of wheelchair badminton players. However, these findings should be interpreted with caution, given that the included performance parameters can be influenced by numerous factors, potentially undermining the robustness of the clustering methodology employed.

This study highlights the need to strengthen the current classification process in wheelchair badminton. To align with IPC guidelines and enhance the methodology employed in this study, integrating functional trunk capacity data could prove to be beneficial.

Acknowledgments

The authors thank the participants in the study as well as the laboratory of the IAPS and the Fédération Française de Badminton.

The authors declare no conflict of interest. This work was supported by a French government grant, managed by the Agence Nationale de la Recherche (ANR) under the "France 2030" program, reference ANR-19-STHP-0005.

References

1. Mann DL, Tweedy SM, Jackson RC, Vanlandewijck YC. Classifying the evidence for evidence-based classification in Paralympic sport. Journal of Sports Sciences. 2021 Aug 13;39(sup1):1–6.

2. Goosey-Tolfrey VL, Leicht CA. Field-Based Physiological Testing of Wheelchair Athletes. Sports Med. 2013 Feb;43(2):77–91.

3. BWF. BWF Statutes, Section 5.5.5: PARA BADMINTON CLASSIFICATION REGULATIONS. BWF. [Internet]. 2020. Available from: https://extranet.bwfbadminton.com/docs/document-system/81/1466/1471/Section%205.5.5%20-%20Para%20Badminton%20Classification%20Regulations.pdf

4. Janiaczyk M. Para-badminton – sport for people with disabilities. Physiotherapy [Internet]. 2015 Jan 1 [cited 2020 Nov 17];23(4). Available from: http://www.degruyter.com/view/j/physio.2015.23.issue-4/physio-2015-0018/physio-2015-0018.xml

5. Bolin I, Bodin P, Kreuter M. Sitting position–Posture and performance in C5–C6 tetraplegia. Spinal Cord. 2000 Jul;38(7):425–34.

6. Gagnon D, Verrier M, Masani K, Nadeau S, Aissaoui R, Popovic M. Effects of Trunk Impairments on Manual Wheelchair Propulsion Among Individuals with a Spinal Cord Injury: A Brief Overview and Future Challenges. Topics in Spinal Cord Injury Rehabilitation. 2009 Sep 29;15(2):59–70.

7. Seelen HAM, Potten YJM, Drukker J, Reulen JPH, Pons C. Development of new muscle synergies in postural control in spinal cord injured subjects. Journal of Electromyography and Kinesiology. 1998 Feb;8(1):23–34.

8. Mota Ribeiro, de Almeida. Performance analysis in wheelchair para-badminton matches. International Journal of Racket Sports Science. 2020;2(1):22–31.

9. Strapasson AM. Are Technical and Timing Components in Para-Badminton Classifications Different? A M. 2021;3:6.

10. Strapasson AM, Chiminazzo JGC, de Almeida MB, Duarte E. Para-badminton: technical and temporal characteristics. 2018;16(2):7.

11. Tweedy SM, Vanlandewijck YC. International Paralympic Committee position stand--background and scientific principles of classification in Paralympic sport. British Journal of Sports Medicine. 2011 Apr 1;45(4):259–69.

12. Bakatchina S, Weissland T, Faupin A. Les dispositifs d'évaluation des parasportifs pratiquant des sports de petits terrains en fauteuil roulant manuel. Benguigui N, Boiché J, Grosprêtre S, Guillot A, Lepers R, Scelles N, et al., editors. Mov Sport Sci/Sci Mot. 2021;(114):55–69.

13. Alberca I, Chénier F, Watier B, Brassart F, Vallier JM, Faupin A. Impact of holding a badminton racket on temporal and kinematic parameters during manual wheelchair propulsion based on forward and backward propulsion. [Internet]. HAL; 2024. Available from: https://hal.science/hal-04594677

14. Rhodes JM, Mason BS, Malone LA, Goosey-Tolfrey VL. Effect of team rank and player classification on activity profiles of elite wheelchair rugby players. Journal of Sports Sciences. 2015 Nov 26;33(19):2070–8.

15. Mason BS, Lenton JP, Goosey-Tolfrey VL. The physiological and biomechanical effects of forwards and reverse sports wheelchair propulsion. The Journal of Spinal Cord Medicine. 2015 Jul;38(4):476–84.

16. van der Slikke RMA, Berger M a. M, Bregman DJJ, Lagerberg AH, Veeger HEJ. Opportunities for measuring wheelchair kinematics in match settings; reliability of a three inertial sensor configuration. J Biomech. 2015 Sep 18;48(12):3398–405.

17. Xu H, Chua JC, Burton M, Zhang K, Fuss FK, Subic A. Development of low cost on-board velocity and position measurement system for wheelchair sports. Procedia Engineering. 2010 Jun;2(2):3121–6.

18. Fuss FK. Speed measurements in wheelchair sports – theory and application. Sports Technology. 2012 Feb;5(1–2):29–42.

19. Poulet Y, Brassart F, Simonetti E, Pillet H, Faupin A, Sauret C. Analyzing Intra-Cycle Velocity Profile and Trunk Inclination during Wheelchair Racing Propulsion. Sensors. 2022 Dec 21;23(1):58.

20. Chénier F. Kinetics Toolkit: An Open-Source Python Package to Facilitate Research in Biomechanics. JOSS. 2021 Oct 23;6(66):3714.

21. Cohen J. Statistical power analysis for the behavioral sciences. 2nd ed. Hillsdale, N.J: L. Erlbaum Associates; 1988. 567 p.

22. Vanlandewijck Y, Theisen D, Daly D. Wheelchair propulsion biomechanics: implications for wheelchair sports. Sports Med. 2001;31(5):339–67.

23. Hintzy-Cloutier F, Tordi N, Rouillon JD, Belli A. The influence of within-cycle pattern characteristics on mechanical parameters developed during wheelchair sprinting? Science & Sports. 2002 May;17(3):128–31.

24. Janssen T, Lenton J, de Baare V, Bakker Y, Bos F, Goosey-Tolfrey V, et al. Acceleration Profile during 20-m Sprints in Elite Wheelchair Tennis Players. 2023;13.

25. Van Der Woude LHV, Drexhage D, Veeger HEJ. Peak power production in wheelchair propulsion. Clinical Journal of Sport Medicine. 1994;4(1):14–24.

26. Veeger HEJ, van der Woude LHV, Rozendal RH. Load on the upper extremity in manual wheelchair propulsion. Journal of Electromyography and Kinesiology. 1991 Dec;1(4):270–80.

27. Gil SM, Yanci J, Otero M, Olasagasti J, Badiola A, Bidaurrazaga-Letona I, et al. The Functional Classification and Field Test Performance in Wheelchair Basketball Players. Journal of Human Kinetics. 2015 Jun 1;46(1):219–30.

28. Tachibana K, Mutsuzaki H, Shimizu Y, Doi T, Hotta K, Wadano Y. Influence of Functional Classification on Skill Tests in Elite Female Wheelchair Basketball Athletes. Medicina. 2019 Nov 15;55(11):740.

 Click here to access/download;Figure;figures.docx

Figure 1

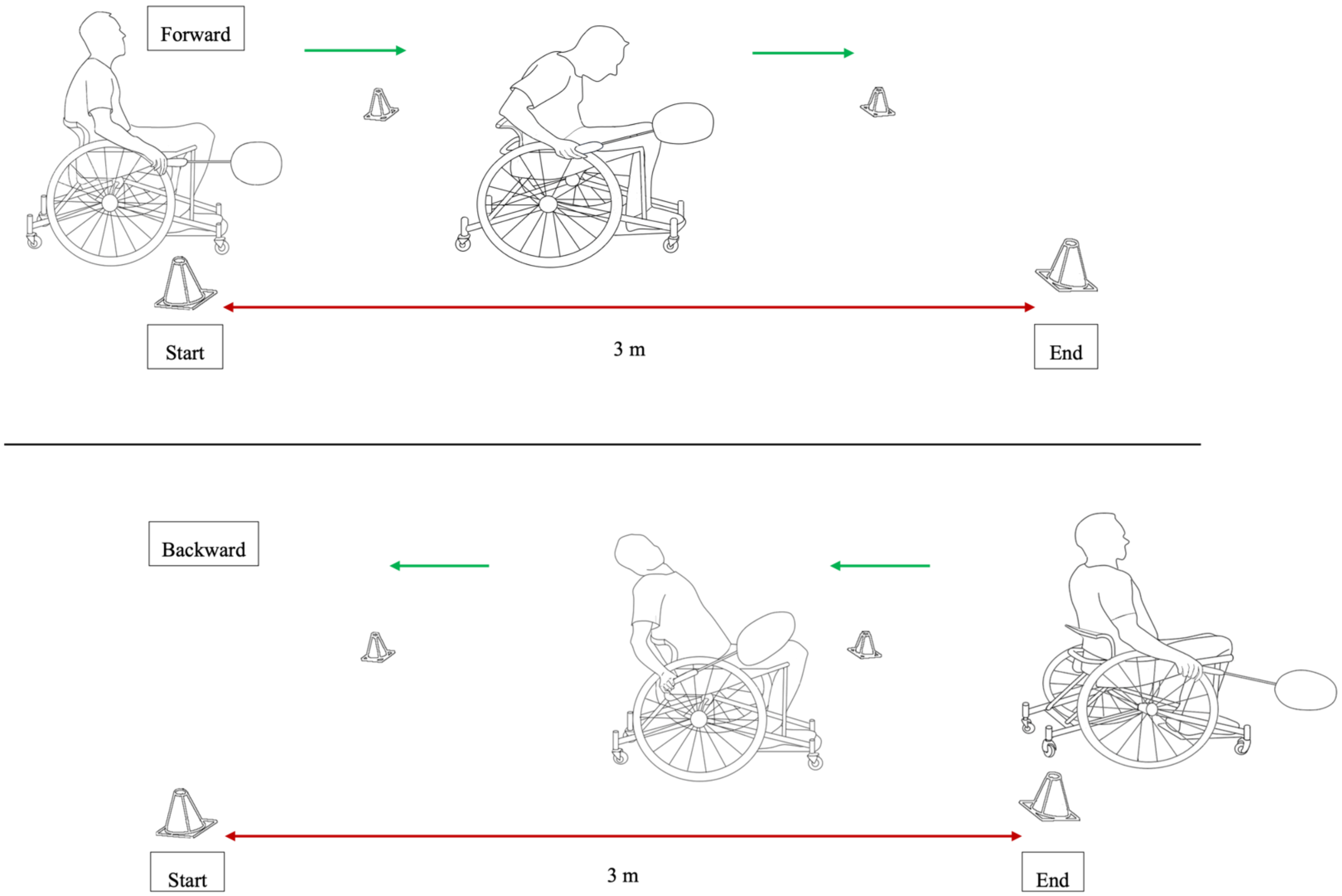

Figure 2

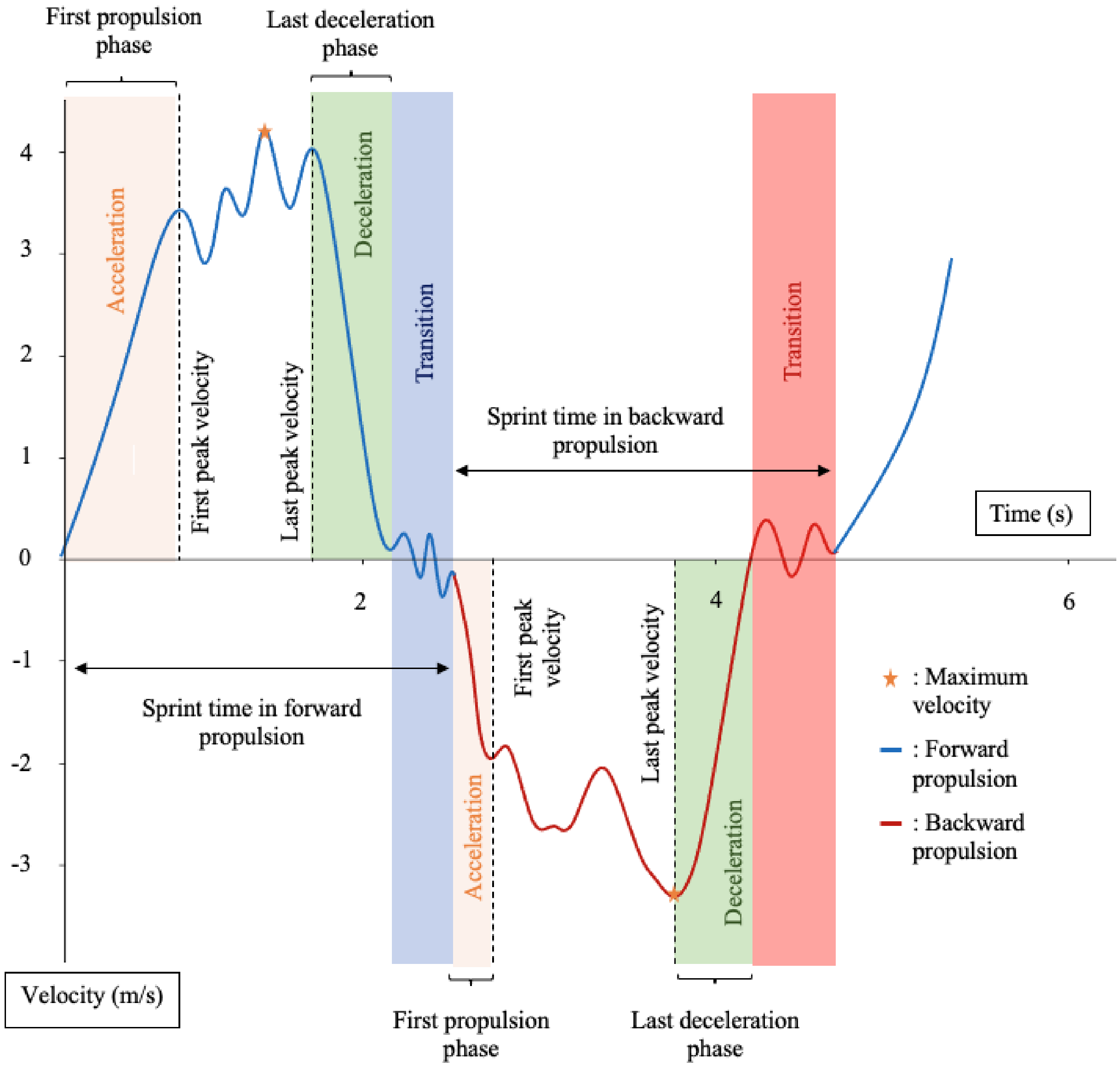
First propulsion phase
Last deceleration phase
Acceleration
First peak velocity
Last peak velocity
Deceleration
Transition
Sprint time in backward propulsion
Transition
Time (s)
Sprint time in forward propulsion
Acceleration
First peak velocity
Last peak velocity
Deceleration
: Maximum velocity
: Forward propulsion
: Backward propulsion
Velocity (m/s)
First propulsion phase
Last deceleration phase
4
3
2
1
0
-1
-2
-3
2
4
6

Figure 3

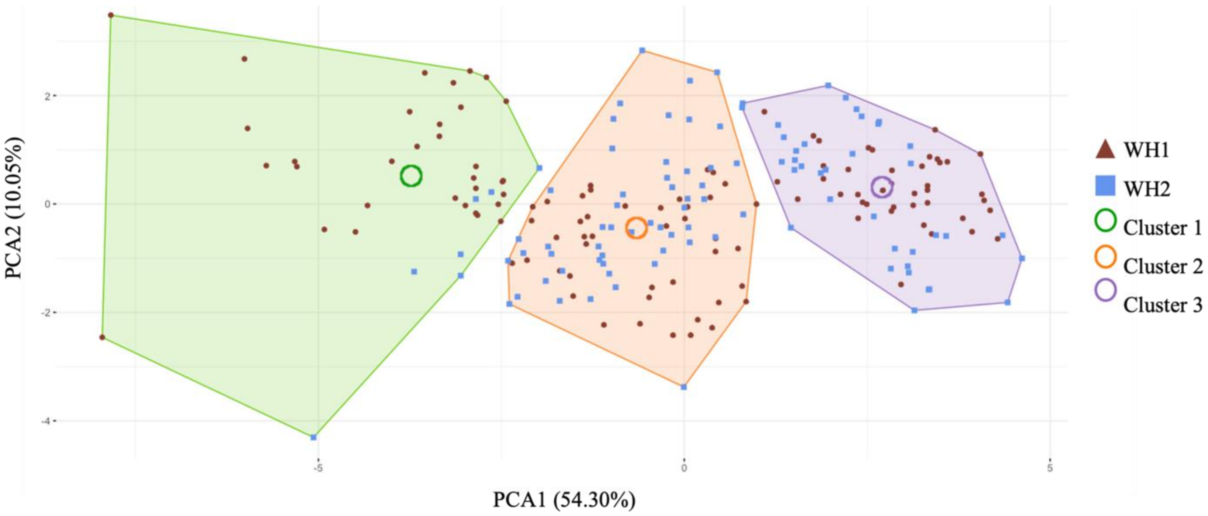

PCA2 (10.05%)
2
0
-2
-4
-5
0
5
PCA1 (54.30%)
WH1
WH2
Cluster 1
Cluster 2
Cluster 3
