# Supplementary material for: Trying to use temporal and kinematic parameters for the classification in wheelchair badminton
Source: PLoS One. 2025 Mar 28;20(3):e0315939. doi: 10.1371/journal.pone.0315939 (PMC11952265; doi:10.1371/journal.pone.0315939)
Supplement: Attachment — Submitted filename: Answers to reviewers_V3.docx [file pone.0315939.s003.docx]

**Answers to reviewers**

**Reviewer 1:**

Dear Authors,

All comments and recommendation you will find in notes in the PDF file.

I hope they are clear for you.

please, highlight all changes in the next version of the manuscript (e.g. blue fond of new parts in the text).

Kind regards,

Dear Jolanta Marszałek,

Thank you very much for your thorough and precise feedback, which has greatly contributed to improving the quality of the article. We have carefully incorporated all your comments and have addressed them in the section below.

Once again, thank you for your valuable work.

Kind regards,

Ilona Alberca

***Abstract:***

Class WH1 and WH2: It’s modified. Thank you.

***Introduction:***

Categories or classes?: Classes more than categories. We changed the vocabulary to avoid being repetitive. However, we unified the terminology. Thank you (l.9).

Athletes: We changed it thank you (l. 9)

Minimal impairment criteria (MIC): We corrected this, thank you (l.14-15).

“or” or “and”?: The word used is ‘or’. The technical assessment can take place during a tournament match or a training match. It depends on the conditions available to the examiner. But both situations can lend themselves to classification.

It would be helpful to point which step of the evidence-based classification approach is Authors’ study (see e.g. Tweedy or Vanlandevijck studies from 2014, 15, 16, 17: We've added the following clarification.

“Based on the study by Tweedy & Vanlandewijck (7), incorporating such parameters into the evidence-based classification process could correspond to ‘Step 3b: Develop measures of (determinants of) performance’ ”.Thank you for your references and advice (l. 40-42).

where IMU was put on an athlete?: IMUs were not placed on the athlete but on the wheelchair wheel hubs. We added that clarification (l. 45). Thank you!

(in bracket inform the reader what is the aim of this one-minute field test).: It's added (l. 47-48).

“forward and backward propulsion test reproducing the movements of wheelchair badminton players”

Thank you.

To delete ‘here’: We deleted that. Thanks.

Was and Past tense? Authors’ study was done, wasn’t it?: Yes indeed, but if you don't mind we'd prefer to keep the future for the introduction.

Truly saying this point should be above explained “However, given their classification based on impairment level, it can be hypothesized that WH1 athletes with more severe functional limitations will demonstrate spatiotemporal patterns indicative of lower performance compared to WH2 athletes”. I mean it should be clearly written what elements classifiers consider athletes’ allocation to groups/class/categories: We added all the details about their classification in the introduction (l. 14-27).

“The classification process is initiated by determining eligibility based on a Minimal Impairment Criteria (MIC), as described by the BWF (3). Once this step is completed, a physical evaluation is conducted, which includes a manual muscle test (5) and/or a joint mobility test (5). The ASIA score should be used for athletes with spinal cord injuries (3,6). Finally, after the physical evaluation, a technical assessment is carried out during a tournament and a training match, where evaluators are asked to identify the following profiles:

-	WH1: "Players exhibit functional limitations based on muscle power and trunk range of motion, and possibly upper limbs, during a match or training session" (3).

-	WH2: "Players have a functional limitation based on reduced muscle power or range of motion, requiring the use of mobility aids. Shifting the center of gravity may result in loss of balance, for example, when attempting to pivot or during stop-and-start movements" (3).

This final assessment may include an evaluation of the player's ability to perform specific tasks and activities that are part of wheelchair badminton (3).”

We hope that will be sufficient. Thanks

In my opinion is rather banal hypothesise: We changed it.

Thus, it can be hypothesized that WH1 athletes with more severe functional limitations will demonstrate temporal and kinematic patterns indicative of lower performance compared to WH2 athletes with lower velocities, acceleration, deceleration and longer sprint time.

Thank you.

Two classifications” or two groups/cathegories/classess?: We unified the term used throughout the article, which is ‘classes’.

***Materials and methods:***

lines 60-62: Is this sentence necesary? “This study aims to explore the possibility that the spatiotemporal data representative of propulsion technique and athlete performance from this study could serve as evidence for the evidence-based classification.”: No indeed, we deleted this sentence. Thank you.

lines 62-63: “The same protocol as in the study by Alberca et al. (13) was used in this study.” it sould be explained in detail and in the end references should be added: This has been added (l. 72-83).

“During the experiment, all athletes started from a stationary position at the 3-meter line, demarcated by cones, initiating in forward propulsion. Subsequently, they braked and proceeded in backward propulsion along the same course, repeating this sequence for 1 min. When switching between propulsion directions, athletes were required to pass the large wheels of their wheelchairs beyond the cones at each end of the track. Two trials were conducted for each participant: one with the racket and one without, with the trial order determined randomly (12). The badminton racket and wheelchair utilized were individualized to each participant and matched those employed in competitions. The athletes' personal wheelchairs featured camber angles ranging from 18° to 20°, with wheel sizes ranging from 24 to 26 inches and a rear anti-tip wheel. Each athlete held the racket on their preferred side, referred to as the racket side. A 5-minute break was kept between each trial. Although the propulsion technique was not prescribed, upon observation, all athletes employed synchronous propulsion.”

Thank you

or abbreviation or full word. Choose one and use it consequently in the whole text: A lack of consistency concerning units indeed. We changed for the abbreviation only (l. 74). Thank you.

Space: Added, thanks (l. 68).

this sentence is obvious :) " Upon the starting signal, they sprinted forward along the 3-meter line until reaching the end”: Indeed! It has been deleted. Thanks.

kept?: We changed “observed” for “kept” (l. 82). Thanks

C - a big letter: Corrected, thanks.

please keep the same writing style of dates: We've changed the style so that it's the same throughout the document (l. 90). Thank you.

weelchair camber: We changed “camber” for “wheelchair camber” (Table 1). Thanks.

health condition: We also changed “pathology” for “health condition” (Table 1).

it should be pointed level of paraplegia: We changed the title and added the level of paraplegia (Table 1). Thank you.

body height/body length: Changed (Table 1). Thanks.

this is not correc calculation

1,62*1,62=2,6244

60/2,6244=22,86: We are sorry for those mistakes. We corrected the BMI calculation. Thank you.

space between number and bracket: It’s corrected (Table 1). Thanks.

above the table: Corrected thanks.

to delete “With”: Deleted.

above the table: Corrected thank you.

In: We changed it.

dot in the end: added.

dot in the end: added.

what is this? “PCA”: This refers to the main component analysis we defined above (l. 212).

Dot: Added.

***Discussion:***

In general:

the discussion is weak because of lack of research results comparison and explanation of Authors’ results: It is true that few comparisons were made with the literature due to the lack of previous research on this topic. However, we have therefore added several sections to the discussion to flesh it out, enable certain comparisons to be made and discuss the results more fully. We hope this will be acceptable (l. 261-265, 277-292, 320-329). Thank you very much!

***Limitation:***

by G-power analysis was done, and there were three more participants than you established in G-power: We modified this part (l. 350-353). Thank you.

**Reviewer 2:**

The paper presents interesting topic of wheelchair badminton athletes classification based on spatiotemporal parameters. The article is well-written, however, the following remarks should be considered:

Dear Reviewer,

Thank you for your positive feedback and for recognizing the relevance of the topic. We will carefully consider and address each of the remarks you have provided to further improve the paper. Thank you.

Kind regards,

On behalf the authors, Ilona Alberca

• The motivation of the study should be indicated more clearly.: WE modified the introduction to be clearer on the motivation of the study. Thank you.

• Please correct dot in the statement “In the initial analysis, significant disparities in velocity were observed between WH1 and WH2 athletes that may have large influence on TTmean”.: The sentence has been corrected. Thank you.

• Adding more clustering methods (like K-Means and Density-based Clustering) would definitely improve the study.: Thank you for your feedback, which prompted a thorough review of this part of the article. The statistical analysis for this study was discussed with statisticians, and considering the nature of the data, how they are utilized, and the study's objective, it was determined that hierarchical clustering was the most appropriate method in this case. Additionally, we consulted with colleagues working on similar objectives who have used the same statistical methods. They also supported the decision to retain the hierarchical clustering analysis.
